# Halotolerant *Gordonia* sp. DH2 enhances hydrocarbon degradation and emulsification in high-salinity oil reservoirs through cell-associated surface-active material

**DOI:** 10.1039/d5ra09392a

**Published:** 2026-04-28

**Authors:** Ji Gao, Yu Duan, Ting Xu, Ding Dong, Dehao Lu, Shuyuan Deng, Jian Fu, Fan Zhang, Hao Dong, Yuehui She

**Affiliations:** a Hubei Engineering Research Centers for Clean Production and Pollution Control of Oil and Gas Fields, College of Chemistry and Environmental Engineering, Yangtze University Jingzhou 434023 China dong_hao2005@163.com; b College of Resources and Environment, Yangtze University Wuhan Hubei 430010 China; c Karamay Xinaoda Petroleum Technological Co., Ltd Xinjiang 83400 China; d Xinjiang Oilfield Company, PetroChina Karamay Xinjiang 834000 China; e College of Petroleum Engineering, Yangtze University Wuhan Hubei 430010 China sheyuehui@163.com; f The Key Laboratory of Marine Reservoir Evolution and Hydrocarbon Accumulation Mechanism, Ministry of Education, College of Energy Resources, China University of Geosciences (Beijing) Beijing 100083 China

## Abstract

*Gordonia* sp. DH2 is a halotolerant hydrocarbon-degrading bacterium isolated from a petroleum reservoir. It effectively degraded alkanes and aromatic hydrocarbons and exhibited strong cell-associated emulsifying activity during hydrocarbon utilization. Whole-genome analysis revealed genes putatively associated with salt tolerance, hydrocarbon degradation and biosurfactant-related functions. The culture conditions affecting emulsifying activity were further investigated. Based on TLC staining and FTIR analysis, the extracted cell-associated surface-active material was inferred to contain carbohydrate-related and lipid-like moieties, suggesting a glycolipid-like surface-active fraction rather than permitting definitive structural identification. Unlike most reported extracellular biosurfactant-producing bacteria, *Gordonia* sp. DH2 exhibited an uncommon cell-associated emulsification phenotype, which enhanced adhesion to hydrophobic surfaces and promoted petroleum emulsification. The strain also showed strong degradation capacity toward both alkanes and aromatic hydrocarbons under saline conditions.

## Abstract 2

*Gordonia* sp. DH2 is a halotolerant hydrocarbon-degrading bacterium isolated from a petroleum reservoir.

## Graphical abstract

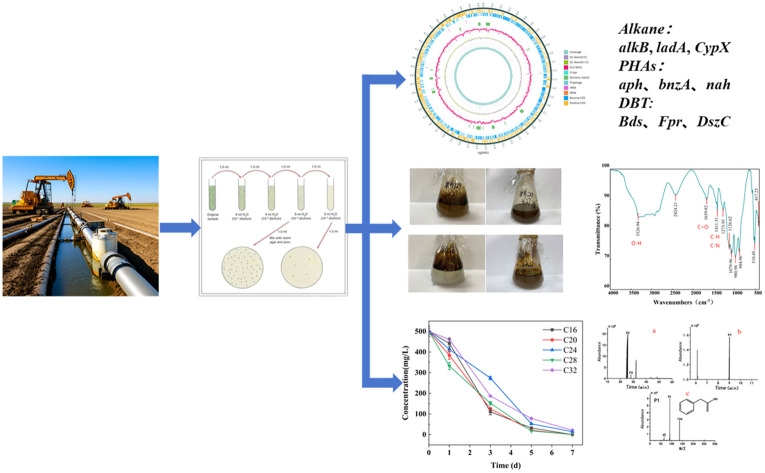

## 1 Introduction

Reservoir environments are often characterized by hydrocarbon-rich organic matter and high salinity, and the effectiveness of bioremediation is frequently limited by such high-salt conditions.^1^ Elevated osmotic pressure induces microbial cell dehydration and enzymatic inhibition, significantly diminishing the efficiency of hydrocarbon degradation. Traditional bioremediation solutions rely on halotolerant strain screening, while most strains are confronted with mass transfer limitations of hydrophobic substrates, as the low water solubility of petroleum hydrocarbons hinders their bioavailability.^2^ The inherently low aqueous solubility of petroleum hydrocarbons further hampers their bioavailability. Although biosurfactants can enhance hydrocarbon solubilization *via* emulsification, secreted biosurfactants such as rhamnolipids produced by *Pseudomonas aeruginosa* are prone to dilution or deactivation through adsorption in complex environmental matrices.^3^

Many oilfield wastewaters are characterized by high salinity and elevated concentrations of petroleum hydrocarbons. Under such extreme conditions, halophilic hydrocarbon-degrading bacteria demonstrate ecological dominance, owing to their capacity to not only survive but also efficiently degrade diverse hydrocarbon substrates (including alkanes and aromatics). The genomes of such strains harbor numerous salt-tolerance genes (such as the Na^+^/H^+^ antiporter gene nhaA and the K^+^ transporter gene kefB), which enable them to maintain osmotic balance under high-salinity conditions.^4^ Zhao *et al.*^5^ previously isolated halophilic microbial consortia from Shengli Oilfield soil, which achieved complete degradation of phenanthrene within 8 days under 5–15% NaCl. Similarly, *Gordonia polyisoprenivorans* ZM27 exhibited robust viability and hydrocarbon-degrading activity under 10% NaCl and nutrient-starved conditions, highlighting the distinct ecological advantage of halophilic hydrocarbonoclastic bacteria in remediating petroleum-contaminated hypersaline environments.

The ability to produce biosurfactants significantly enhances the bioremediation efficacy of hydrocarbon-degrading bacteria. However, not all such bacteria possess biosurfactants-producing capabilities. Several species within the *Gordonia* sp. have been identified as efficient biosurfactants producers, capable of effectively degrading hydrocarbons in petroleum-contaminated matrices. For instance, *Gordonia* sp. BS29, isolated from diesel-contaminated soils, produces two structurally distinct biosurfactants.^6^*Gordonia* sp. IITR100, cultivated under petroleum hydrocarbon-enriched conditions, secretes glycolipid-type biosurfactants that reduce the surface tension of aqueous media from 61.06 mN m^−1^ to 36.82 mN m^−1^, thereby enhancing hydrocarbon emulsification and uptake.^7^ The strain demonstrated substantial biosurfactants production and notable efficiency in hydrocarbon degradation.

It is noteworthy that certain bacterial strains do not secrete biosurfactants extracellularly, but rather anchor them to the cell surface, forming a self-emulsifying interfacial layer, or partially retain them at the cell envelope.^8,9^ This cell-associated mode of biosurfactants localization minimizes surfactant loss and facilitates direct bacterial interaction with hydrophobic substrates. However, the underlying molecular mechanisms governing biosurfactants retention in halophilic strains remain poorly characterized. Moreover, the behavior and functional mechanisms of biosurfactants differ significantly depending on their localization—either dissolved in the aqueous phase or adsorbed onto the cell surface.^10–13^ When attached to the cell membrane, biosurfactants may alter cell surface hydrophobicity *via* physical adsorption or covalent binding. Nonionic surfactants, for example, can enhance hydrophobicity by forming a coating on the bacterial surface. Additionally, certain biosurfactants promote biofilm formation, further modifying surface characteristics. In the context of hydrophobic carbon source utilization (*e.g.*, crude oil), soluble biosurfactants primarily function by reducing oil–water interfacial tension and forming oil-in-water (O/W) emulsions, thereby indirectly enhancing microbial access to hydrocarbons. In contrast, cell-bound biosurfactants can facilitate emulsification at the immediate interface, breaking down oil droplets into stable and smaller droplets and increasing the contact surface area between cells and oil droplets, and consequently improving substrate bioavailability and degradation efficiency.

In this study, *Gordonia* sp. DH2 was isolated from oilfield production water and exhibited stable growth under high salinity and high mineralization. We investigated both the biosurfactants-producing potential of *Gordonia* sp. DH2 and the environmental factors influencing its emulsifying activity synthesis. The cell-associated surface-active materials were structurally characterized using Fourier Transform Infrared Spectroscopy (FTIR), and their emulsification mechanisms were analyzed based on cell surface hydrophobicity. Furthermore, using petroleum hydrocarbons and crude oil as the sole carbon and energy sources, the hydrocarbon degradation capacity of strain DH2 was assessed. Gas Chromatography-Mass Spectrometry (GC-MS) was employed to quantify degradation rates of aliphatic and aromatic hydrocarbons and to evaluate the strain's crude oil biodegradation efficiency.

## 2 Materials and methods

### 2.1 Microbial materials

The strain *Gordonia* sp. DH2 was isolated by our laboratory from the Daqing Oilfield and is preserved in the China Center for Type Culture Collection with the accession number CCTCC 20241126. The biosurfactant-producing strains used in this work-including *Pseudomonas aeruginosa* PAO1 (ATCC 15692), *Bacillus subtilis* W-2 (CGMCC 9985), and *Brevibacillus borstelensis* YZ-2 (CGMCC 9981)-were either maintained in our laboratory or purchased from culture collections, as referenced.^14,15^

### 2.2 Whole genome sequencing

DNA extraction was performed using a commercial kit (QIAGEN, Hilden, Germany) and confirmed *via* agarose gel electrophoresis and Nanodrop analysis for purity assessment.^16^ Upon passing the quality control tests, the extracted DNA underwent damage repair and end repair procedures. Following magnetic bead purification, barcode labels were attached to the DNA ends using the NBD104 and NBD114 kits (Nanopore, Oxford, England). Another round of magnetic bead purification was conducted before ligating sequencing adapters from the library preparation kit. The DNA library was then quantified using a Qubit fluorometer to ensure precise concentration measurement.

The prepared DNA library, at the specified concentration and volume, was loaded onto a flow cell and transferred to the PromethION sequencer for real-time single-molecule sequencing. After sequencing, the raw data were subjected to quality assessment and subsequent bioinformatics analysis.

### 2.3 Salinity tolerance of strain DH2

Extra concentrations of NaCl (2 g L^−1^, 4 g L^−1^, 6 g L^−1^, 8 g L^−1^, 10 g L^−1^, 12 g L^−1^) and 2 g L^−1^ sucrose were added to the basal medium (NaNO_3_ 10, Na_2_MoO_4_ 0.08, MgSO_4_ 0.2, KH_2_PO_4_ 1, (NH_4_)_2_HPO_4_ 1, g L^−1^, pH 7.0–7.2), with all other components and concentrations remaining unchanged. The pH was maintained at 7.2, and the cultures were incubated at 150 rpm for 3 days. The OD_600_ value was subsequently measured to assess microbial growth.

### 2.4 Impact of water-soluble carbon sources and hydrocarbons on biosurfactant synthesis

Various carbon sources were incorporated into the basal medium, with all other components and concentrations remaining unchanged. The pH of the medium was maintained at 7.2, and the cultures were incubated at 150 rpm for 7 days. The carbon sources tested included petroleum hydrocarbons (500 mg L^−1^ respectively): naphthalene, anthracene, phenanthrene, pyrene, *n*-hexadecane (C16), eicosane (C20), tetracosane (C24), octacosane (C28), dotriacontane (C32), crude oil, liquid paraffin, and solid paraffin; and non-petroleum hydrocarbons (10 g L^−1^ respectively): sucrose, glucose, starch, and sodium acetate. The bacterial strains were then inoculated, and the cultures were incubated at 37 °C for 4 days. Following incubation, the OD_600_ value, emulsification index, and surface tension were measured.

### 2.5 Emulsification index (*E*_24_) and OD_600_

The emulsification index was determined using the method outlined by Yin *et al.*, with slight modifications.^17^ A 3 mL aliquot of culture supernatant was mixed with an equal volume of liquid paraffin (1 : 1 v/v), allowed to stand for 24 hours, and then the height of the emulsion layer and the total liquid height were measured according to eqn (1).1
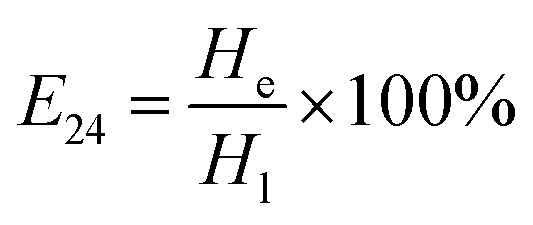
where *H*_e_ and *H*_l_ denote the height of the emulsified layer and the height of the liquid layer, respectively.

And the OD_600_ value was measured at 600 nm wavelength using a UV-vis spectrophotometer with deionized water as blank.

### 2.6 Extraction and characterization of the cell-associated surface-active material

Following culture, the broth was centrifuged and the pelleted biomass was washed twice with sterile ultrapure water prior to lyophilization. The cell-associated surface-active material was then extracted following the protocol of Cui *et al.*^18^ Specifically, the lyophilized cells were reconstituted in deionized water, followed by solvent extraction using a dual-phase system of CHCl_3_/CH_3_OH (1 : 2, v/v) at a twofold volumetric ratio. The organic phase was carefully collected, evaporated to complete dryness under reduced pressure, and further fractionated *via* column chromatography. Fractions demonstrating significant emulsification potential were pooled for downstream analytical characterization.

The silica gel-purified product was spotted onto a silica gel TLC plate. The mobile phase used was chloroform : methanol : water (7 : 3 : 1, v/v/v). After development, the TLC plate was sprayed with colorimetric reagents for visualization: ninhydrin staining to detect peptides and amino acids, and *p*-anisaldehyde staining to identify sugars.^19^

To identify the functional groups and bonding types in the extracted surface-active material, Fourier-transform infrared (FTIR) spectroscopy was performed. The spectra were recorded in the Attenuated Total Reflectance (ATR) mode over a wavenumber range of 400–4000 cm^−1^.

### 2.7 Cell-associated surface-active material effects on bacterial emulsification and hydrophilicity/hydrophobicity

To verify the uniqueness of the cell-associated surface-active material produced by the strain *Gordonia* sp. DH2, the laboratory's existing strains *Pseudomonas aeruginosa*, *Bacillus subtilis* and *Brevibacillus borstelensis* were cultured following the same method. After centrifugation to remove the supernatant, the pellets were resuspended to obtain pure bacterial suspensions. These were then compared with the strain *Gordonia* sp. DH2 in terms of their emulsifying properties.

Hydrophilic and hydrophobic quartz sand surfaces were prepared using Karen's method to investigate bacterial cell adsorption.^20^ Bacterial cells were washed three times with ultrapure water, collected by centrifugation at 12 000 rpm for 2 min, and resuspended in ultrapure water to measure the OD_600_. Then, 3 mL of bacterial suspension was added to 10 mL centrifuge tubes containing 1 g each of hydrophilic and hydrophobic quartz sand, followed by incubation in a shaking incubator at 37 ± 1 °C for 3 h. After settling, the OD_600_ of the supernatant was measured. The static adsorption rate of bacterial cells on hydrophilic and hydrophobic quartz sand surfaces was calculated using the following eqn (2):2
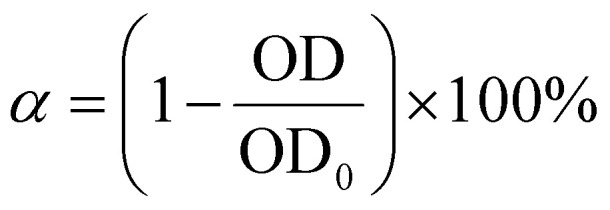
where *α* represents the adsorption rate (%); OD denotes the optical density of bacterial cells in the supernatant after adsorption equilibrium; OD_0_ indicates the optical density of bacterial cells in the supernatant before adsorption equilibrium.

### 2.8 Emulsion stability studies

The stability of the surface-active material was evaluated using *E*_24_ (emulsification index after 24 h) as a stability indicator.^21^

#### 2.8.1 Substrate emulsification ability

The extracted material was mixed with kerosene, liquid paraffin, crude oil, *n*-heptane, diesel oil, and soybean oil at a 1 : 1 volume ratio. The mixture was left to stand for 24 hours, and *E*_24_ was calculated.

#### 2.8.2 Thermal stability

The biosurfactant was thoroughly mixed with liquid paraffin and stored in a 10 mL stoppered test tube. The samples were heated in a water bath at 40 °C, 50 °C, 60 °C, 70 °C, 80 °C, and 90 °C for 2 hours. After incubation, the mixtures were left to stand, and *E*_24_ was measured at different time points.

#### 2.8.3 pH sensitivity

The pH of the surface-active material solution was adjusted to 2, 3, 4, 5, 6, 7, 8, 9, 10, 11, and 12. Each solution was mixed with liquid paraffin, vigorously shaken, and left to stand. *E*_24_ was measured at different time intervals.

#### 2.8.4 Salinity tolerance

To investigate the effect of salinity on emulsifying activity, NaCl and CaCl_2_ were added separately. Surface-active material solutions with NaCl and CaCl_2_ concentrations of 5%, 10%, 15%, 20%, 25%, 30%, and 35% were prepared. Each solution was mixed with liquid paraffin, vigorously shaken, and left to stand. *E*_24_ was measured at different time intervals to assess the impact of NaCl and CaCl_2_ concentrations on emulsification activity.

### 2.9 Degradation of hydrocarbons by DH2 under high salinity

The carbon source in the culture medium was systematically replaced with individual *n*-alkanes (C16, C20, C24, C28, and C32; 500 mg L^−1^ each), polycyclic aromatic hydrocarbons (naphthalene, anthracene, phenanthrene, and pyrene; 200 mg L^−1^ each), or dibenzothiophene (DBT; 100 mg L^−1^). All experiments were conducted in triplicate under 6% (w/v) NaCl, with all other medium components unchanged. Strain DH2 was inoculated at 5% (v/v), and uninoculated sterile controls were prepared in parallel for each hydrocarbon substrate to account for abiotic loss during incubation and extraction. Cultures were incubated at 37 °C and 170 rpm for 7 days. After incubation, residual hydrocarbons were extracted and quantified by GC-MS. For *n*-alkanes, samples were extracted with *n*-hexane (3 × 10 mL) containing hexadecane-d_34_ as an internal standard (0.2076 mg mL^−1^, 50 µL). For PAHs, samples were acidified and extracted with dichloromethane (3 × 5 mL) containing anthracene-D10 as a surrogate standard (0.132 mg mL^−1^, 50 µL). For DBT, samples were extracted with ethyl acetate containing 4-methylDBT as an internal standard (0.12 mg mL^−1^, 50 µL). Hydrocarbon quantification was performed using a standard calibration eqn (3).^22^3
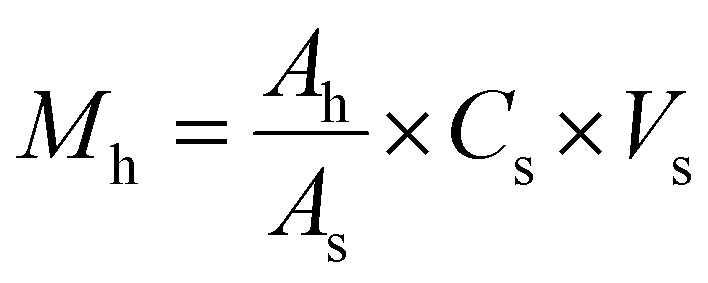
where *M*_h_: the mass of the compound (mg); *A*_h_: the total area of the compound; *A*_s_: the integrated area of the standard sample; *C*_s_: the concentration of the standard sample (mg ml^−1^); *V*_s_: the volume of the standard sample (0.05 mL).

The degradation rate was calculated by comparing the residual amount of each hydrocarbon in the inoculated cultures with that in the corresponding sterile control after the same incubation period, using the following eqn (4):4
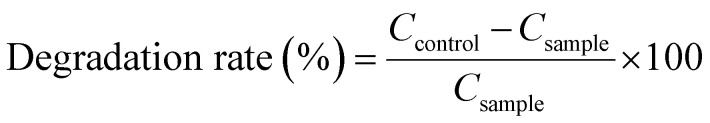
where *C*_control_ is the residual hydrocarbon concentration in the sterile control and *C*_sample_ is the residual hydrocarbon concentration in the inoculated culture.

## 3 Results and discussion

### 3.1 Genome sequencing

#### 3.1.1 Salinity tolerance

The sequencing depth distribution and general genome features are shown in Fig. S1a and Table S1. The genome of *Gordonia* sp. DH2 consists of a circular chromosome of 5 054 758 bp with a GC content of 67.38%. Genomic analysis of *Gordonia* sp. DH2 detected NaCl tolerance and identified genes encoding Na^+^/H^+^ antiporters and K^+^/Cl^−^ transporters, including *nhaA*, *kefB*, *araJ*, and *fucP* (Table S2). *Gordonia* sp. have been isolated from high-salinity soils and aquatic environments, demonstrating remarkable salt tolerance.^23–25^*Gordonia* sp. have evolved to saline conditions by developing specific osmoregulatory mechanisms. They enhance adaptation to highly mineralized environments through regulation of key osmolyte synthesis genes and ion transport systems.^4^

The primary mechanism for salt tolerance is the strain's ability to effectively regulate Na^+^ and H^+^ concentrations. The *nhaA* gene present in strain DH2 encodes an important Na^+^/H^+^ antiporter, which helps maintain the ion balance between the intracellular and extracellular environments by exchanging Na^+^ from the outside with H^+^ from the inside.^26–28^ The *kefB* gene, which encodes an important K^+^/H^+^ exchanger, likely regulates the intracellular potassium ion concentration and may work in coordination with other similar ion transporters (such as *nhaA*) to jointly maintain ion homeostasis, acid–base balance, and osmoregulation, enabling the bacterium to survive in high-mineralization, high-osmotic-pressure environments.^29,30^

#### 3.1.2 Degradation of petroleum hydrocarbons

Genes encoding functions for hydrocarbon degradation and biodesulfurization, such as *alkB*, *catA*, *SfnG*, *ladA*, *Bds*, *pcaC*, *fadA*, and *fadI* (Table 1), were also identified in the DH2 genome.

**Table 1:** Hydrocarbon-degrading genes within the genome of strain DH2

Type	Main enzyme	Genes	Primary function	Involved pathway
Alkane	Alkane monooxygenase	*alkB*, *ladA*, *CypX*	Oxidizes alkanes to primary alcohols	Short-, medium-, and long-chain alkane degradation
	Alcohol/aldehyde dehydrogenase	*adh*, *ald*, *fpr*	Converts alcohols into fatty acids	Intermediate step in alkane degradation
	Long-chain acyl-CoA synthetase	*fadD13*, *fadK*	Activates fatty acids into acyl-CoA	Long-chain alkane degradation
	β-Oxidation key enzymes	*fadA*, *fadB*, *MabA*	Decompose acyl-CoA into acetyl-CoA	Final steps of all alkane degradation pathways
	Cytochrome P450 oxidase system	*CypX*, *rubredoxin*	Assists hydroxylation of alkanes	Medium- and long-chain alkane degradation
	Regulatory proteins	*EAL*, *GGDEF domain proteins*	Involved in c-di-GMP signal regulation	Regulation of alkane degradation pathways
PAHs	Aromatic hydrocarbon monooxygenase	*YrpB*, *SsuD*	Catalyzes the initial hydroxylation of PAHs, converting aromatic hydrocarbons into phenols or alcohols	Initial oxidation stage
	Aromatic hydrocarbon dioxygenase	*aph*, *bnzA*, *nah*	Promotes aromatic ring cleavage and generates *cis*-dihydrodiol intermediates	Ring-cleavage stage
	Oxidoreductase	*Fpr*	Transfers electrons to maintain the redox activity of the oxidation system	Electron transfer stage
	DBT 4S pathway	*Bds*, *Fpr*, *DszC*	Functions analogously to Dsz enzymes to catalyze DBT desulfurization reactions	DBT degradation stage

During the initial oxidation stage of alkane degradation, alkanes undergo hydroxylation to form alcohols-a reaction catalyzed by an alkane hydroxylase complex consisting of AlkB, LadA, and CypX enzymes in *Gordonia* sp. DH-2. Among these, AlkB and CypX are primarily active on medium-chain alkanes (C8–C16). The alkB gene encodes a membrane-bound non-heme di-iron alkane hydroxylase (AlkB), an enzyme family predominantly active on alkanes longer than C10.^31,32^ Research on enzymes and genes involved in the hydroxylation of long-chain alkanes (C15–C36) remains relatively limited. The known oxygenases capable of long-chain alkane hydroxylation mainly include the flavin-dependent monooxygenase encoded by almA and the long-chain alkane degradation enzyme encoded by ladA.^33,34^ Currently, the almA gene cluster has been identified in microorganisms such as *Acinetobacter*, *Salinisphaera*, *Parvibaculum*, *Alcanivorax*, *Marinobacter*, among others. Of these, *Alcanivorax* and *Marinobacter* are recognized as the genera most frequently found to carry the almA gene.^35^ In the genome of strain DH2, the long-chain alkane degradation gene identified was ladA, whereas almA was not detected. LadA encodes a flavin-dependent monooxygenase that specifically binds long-chain alkanes (C15–C36) and catalyzes their terminal hydroxylation using a C4a-hydroperoxyflavin intermediate. This gene was first identified and characterized in the genome of *Geobacillus thermodenitrificans* NG80-2, isolated from the Dagang Oil Field.^33^ To date, the reported distribution of ladA has been mainly restricted to a limited number of thermophilic and oil-tolerant microbial groups, predominantly within the genus *Geobacillus*. Overall, microorganisms reported to carry ladA remain relatively scarce, and few studies have reported its presence in bacteria of the genus *Gordonia*.

PAHs are a part of petroleum hydrocarbons that are difficult to degrade. The genes in DH2 such as *aph*, *bnzA*, *nah*, *Fpr*, *fadA*, and *fadI* (Table 1) are required for the PAH degradation pathway.^36–38^ Initially, aromatic monooxygenases and dioxygenases such as *aph*, *bnzA* and *nah* introduce oxygen atoms into the aromatic rings, converting compounds like naphthalene, anthracene, phenanthrene, and DBT into dihydroxylated intermediates such as *cis*-dihydrodiols. These intermediates are then dehydrogenated to form catechols or other dihydroxy-aromatic compounds, which undergo ring-cleavage catalyzed by catechol dioxygenases to yield open-chain products such as muconic acid derivatives. For sulfur-containing PAHs such as DBT, a specific 4S desulfurization pathway operates, in which DBT is sequentially oxidized to DBT sulfone by monooxygenases and then desulfurized to 2-hydroxybiphenyl by desulfinase-like enzymes, releasing sulfur as sulfite.^39,40^*Bds* and *DszC* found in the genome encode enzymes or cofactors involved in the metabolic transformation of sulfur. The *Bds* gene plays a critical role in the biodesulfurization of benzothiophene (BT),^41^ where a series of oxidation and desulfurization reactions convert BT into 2-hydroxybiphenyl and sulfate.

The distribution of the *Bds* genes is currently mainly restricted to the order Actinomycetales, including the genera *Rhodococcus*, *Gordonia*, *Mycobacterium*, and *Nocardia*. These genes represent the characteristic functional markers of the 4S biodesulfurization pathway for organic sulfur compounds in petroleum. The presence of *Bds* genes suggests *Gordonia* sp. DH2 to mineralize refractory organosulfur compounds into sulfate, thereby providing an accessible inorganic sulfur source for the environment and enhancing its survival capability in crude oil and sulfur-contaminated soils. Given that *Gordonia* sp. generally exhibit strong cell surface hydrophobicity, a high adhesion capacity, efficient biofilm formation, and strong tolerance to various toxic hydrocarbons in oil-polluted habitats, the incorporation of the 4S pathway further expands the metabolic substrate spectrum of DH2, enabling it to simultaneously accomplish alkane degradation and organosulfur removal.^42,43^

### 3.2 Salinity tolerance of strain DH2

**Fig. 1:**
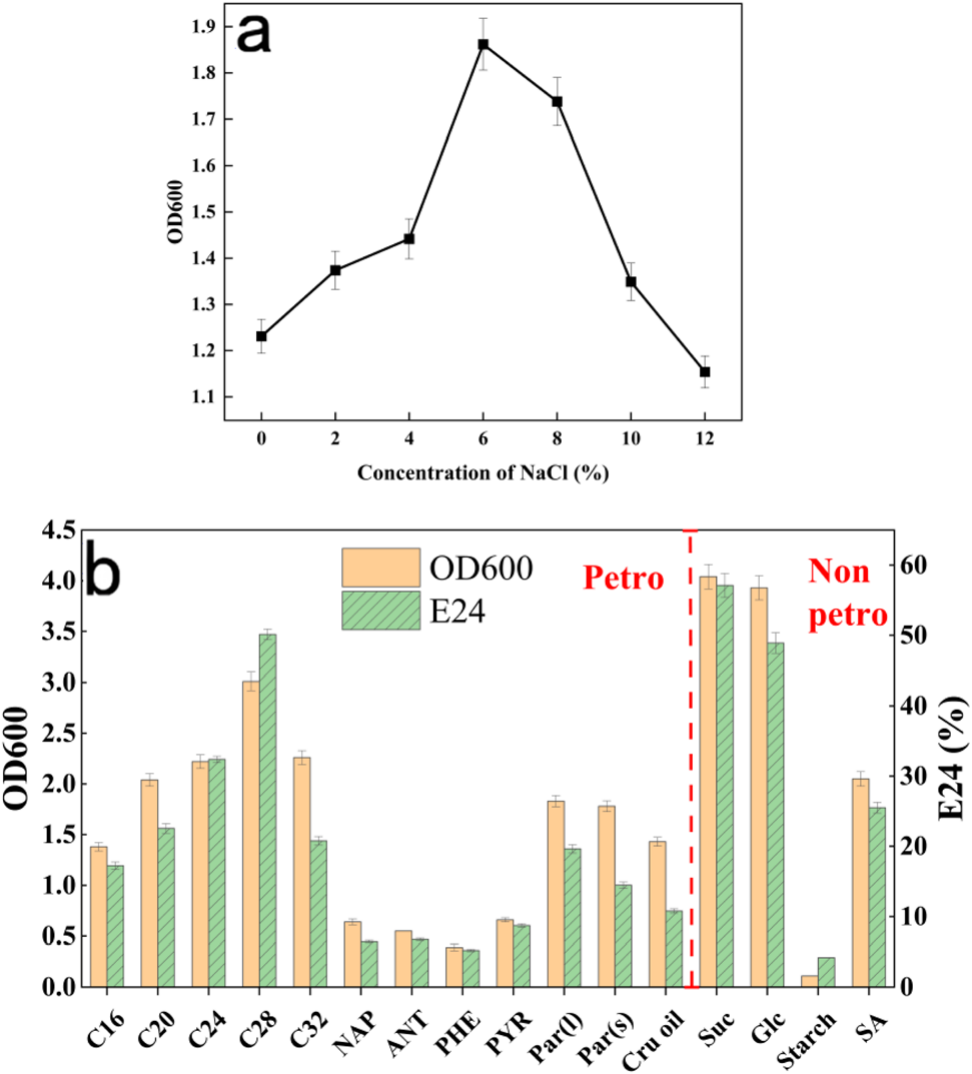
(a) Growth of the strain DH2 under different NaCl concentration conditions; (b) petroleum hydrocarbon carbon sources and non-petroleum hydrocarbon carbon sources.

Following 3 days' cultivation, the strain demonstrated optimal growth at 6% NaCl (OD_600_ = 1.862, Fig. 1a), while maintaining robust growth across the entire tested range (2–12%) compared to the NaCl-free control. Remarkably, even at the highest salinity (12%), no growth inhibition was observed, thereby confirming its exceptional halotolerant capacity. Furthermore, the data suggested that specific NaCl concentrations might potentially enhance bacterial growth.

Growth of *Gordonia* DH2 was observed under hypersaline conditions. Similarly, tolerance to saline concentrations exceeding 10% NaCl has been documented in *Gordonia alkanivorans* CGMCC6845.^44^ These collective findings suggest that salt-resistance genes encoded within *Gordonia* sp. genomes were functionally expressed. However, such expression was confined to discrete *Gordonia* sp. and cannot be generalized across the genus, as it is intrinsically linked to environmental provenance, genetic determinants, and transcriptional regulation mechanisms. From a mechanistic perspective, genomic analyzes reveal that *Gordonia* sp. primarily achieve osmotic adaptation through specialized genetic systems responsible for compatible solute biosynthesis and transport. More precisely, the encoded enzymes and transporter proteins collaboratively regulate osmolyte production and accumulation, thereby maintaining intracellular osmotic homeostasis under hypersaline conditions. Consequently, such inherent halotolerant properties position *Gordonia* sp. as a promising candidate for environmental bioremediation and industrial applications under extreme saline conditions.

### 3.3 Synthesis of surface-active material using hydrocarbons as the sole carbon source

The experimental design incorporated two categories of carbon sources – petroleum hydrocarbons and non-petroleum compounds – to evaluate their distinct effects on bacterial growth and surface-active material production.

For non-petroleum hydrocarbons (Fig. 1b), sucrose yielded maximal growth (OD_600_ = 6.04) and emulsification (*E*_24_ = 57%) with concurrent surface tension reduction to 40.3 mN m^−1^ (Fig. S3). As a disaccharide, sucrose-along with the monosaccharide glucose-proved to be preferentially metabolized by DH2 for surface-active material synthesis, indicating the strain's affinity for structurally simple sugars. Growth measurements (OD_600_) were obtained for five alkane substrates (Fig. 1b), with octacosane (C28) proving most favorable for strain DH2, yielding maximum biomass (OD_600_ = 3.01) and emulsification activity (*E*_24_ = 50%), along with reduced surface tension (58.8 mN m^−1^, Fig. S3). These results confirm strain DH2's ability to utilize petroleum hydrocarbons exclusively for both growth and surface-active material synthesis. Visible emulsion formation demonstrated remarkable emulsifying capability, correlating with increased cell surface hydrophobicity. Earlier research by Mu *et al.*^45^ reported similar hydrophobicity enhancement in bacteria grown with hexadecane. Meanwhile, in this study, aromatic hydrocarbons, crude oil, and paraffins showed varying degrees of emulsification. This demonstrates how hydrophobic carbon sources stimulate DH-2 surface-active material generation, thereby improving both culture growth and PAH degradation efficiency in liquid media.

Numerous microorganisms demonstrate the capability to synthesize biosurfactants utilizing diverse carbon sources. For instance, *Pseudomonas aeruginosa* utilizes lipids to produce rhamnolipids, while *Bacillus subtilis* synthesizes lipopeptides from substrates such as starch and sugars.^14,46^ Significantly, strain DH2 exhibits the capacity for concomitant utilization of both these carbohydrates and petroleum hydrocarbons, demonstrating excellent biosynthetic performance. This confers enhanced adaptability to diverse carbon source types.

### 3.4 Surface-active material characterization

**Fig. 2:**
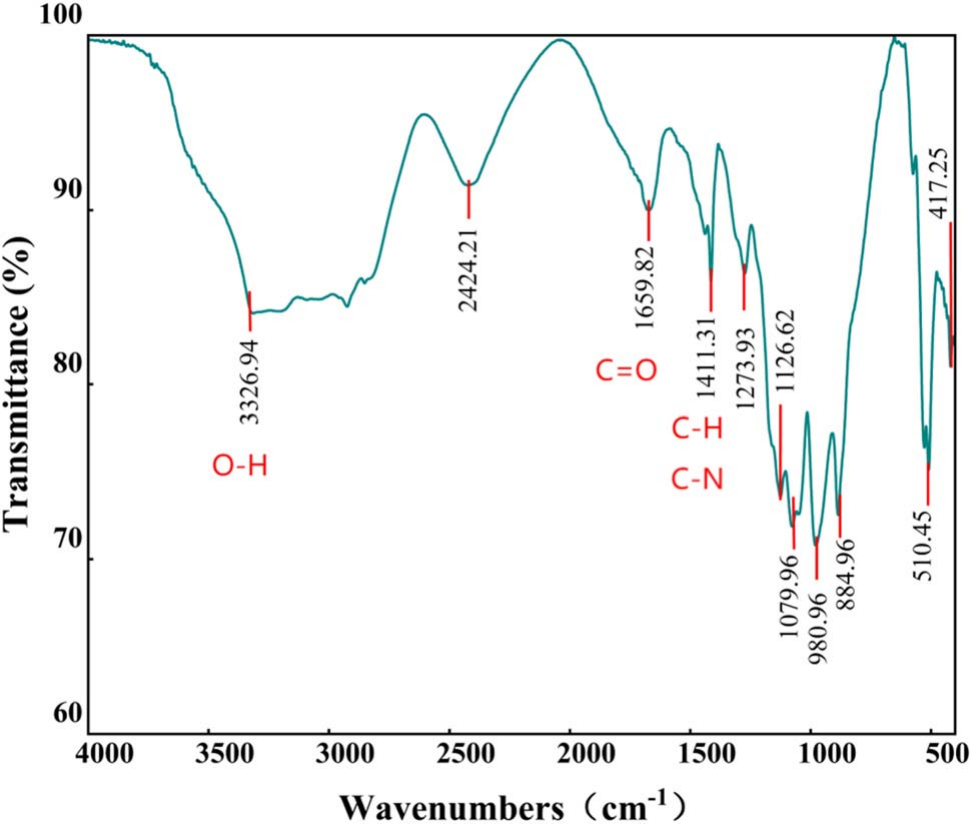
FTIR spectrum of surface-active material produced by *Gordonia* sp. DH2.

When the thin-layer chromatography (TLC) plate was stained with ninhydrin and *p*-anisaldehyde, the *p*-anisaldehyde test yielded a positive result while the ninhydrin test showed negative, which suggests that the surface-active material contain sugar but no amino acids. Fourier-transform infrared spectroscopy (FTIR) analysis was conducted to identify the main functional groups of the surface-active material produced by strain DH2 using the petroleum hydrocarbon octacosane as the sole carbon source.^7,47,48^ The FTIR spectrum (Fig. 2) revealed several characteristic peaks: a broad band at 3326.94 cm^−1^ corresponding to O–H stretching vibrations; a sharp peak at 1659.82 cm^−1^ representing C=O stretching vibrations, suggesting the possible presence of –COO– groups; a peak at 1411.31 cm^−1^ indicating C–H bending and C–N stretching vibrations; and a weak absorption at 1079.96 cm^−1^, suggesting the structure was primarily composed of fatty acids or fatty alcohols. Two additional peaks appeared in the 900–500 cm^−1^ range (510.45 cm^−1^ and 417.25 cm^−1^), vibrations typically associated with long-chain alkanes that may indicate complex fatty structures. Collectively, these spectral features suggest the surface-active material may be consist of long-chain aliphatic compounds, with peaks at 1411.31 cm^−1^ and 883.22 cm^−1^ suggesting the presence of long-chain alkane or fatty acid structures characteristic of octacosane metabolites. TLC staining suggested the presence of carbohydrate-related components in the extracted fraction, while FTIR analysis revealed several characteristic functional groups, including hydroxyl, carbonyl and aliphatic-chain-related signals. These results indicate that the extracted cell-associated surface-active material possesses amphiphilic characteristics and likely contains both carbohydrate-related and lipid-like moieties. Therefore, the product may represent a glycolipid-like substance. However, TLC and FTIR alone are insufficient for definitive structural identification, and more rigorous analyses such as LC-MS, GC-MS or NMR will be required in future studies to clarify its exact composition and structure.

### 3.5 Emulsion stability studies

**Fig. 3:**
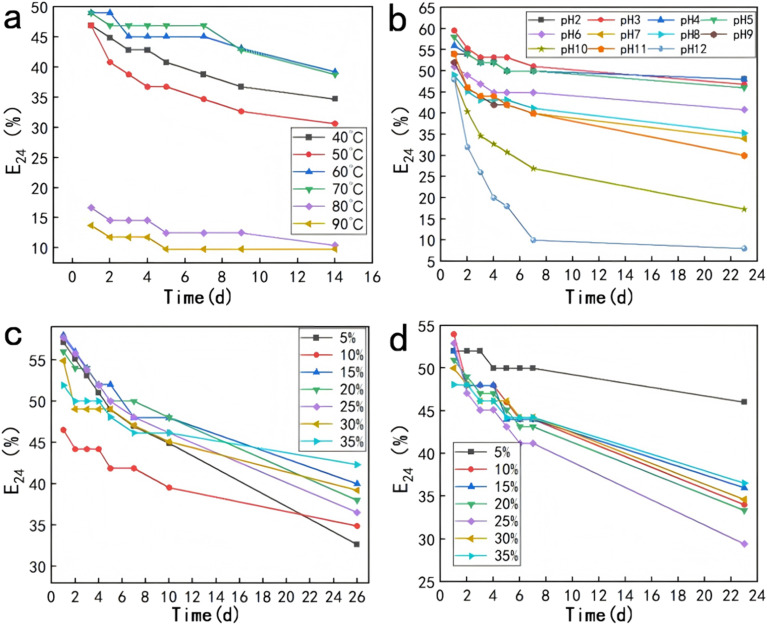
Factors influencing biosurfactant emulsion stability (a) effect of temperature; (b) effect of pH; (c) effect of NaCl concentration; (d) effect of CaCl_2_ concentration.

The emulsion stability studies revealed that the emulsion produced exhibited emulsifying activity towards non-water-soluble organic compounds such as liquid paraffin, crude oil, and soybean oil, indicating that the surface-active material can enhance the solubility of these non-water-soluble organics, thereby improving the degradation efficiency of organic compounds in the environment. The emulsifying activity remained stable within the temperature range of 40 °C to 70 °C (Fig. 3a). However, at 80 °C, the emulsification index decreased by nearly half. Under acidic conditions, the emulsion remained relatively stable, whereas under alkaline conditions, the stability was the lowest. The emulsification index decreased significantly over 7 days, from 41.76% at pH 7 to 9.87% at pH 12 (Fig. 3b). Kazemzadeh *et al.*^49^ previously indicated that increasing salt concentration may lead to a decrease in emulsion stability. However, in this study, increasing the salt concentration up to 35% (Fig. 3c) did not significantly affect the emulsifying activity, with the emulsification index remaining stable at *E*_24_: 40–58%. The emulsion's emulsifying activity remained stable under NaCl concentrations ranging from 5% to 35% (Fig. 3d) for 10 days, and only showed a notable decline after 26 days (*E*_24_: 30–42%).

Similarly, emulsions demonstrated stable emulsification activity under CaCl_2_ concentrations ranging from 5% to 35% and maintained good emulsifying activity for 10 days. Therefore, this study demonstrates that the emulsion exhibits excellent salt tolerance and retains its stability over an extended period, enabling it to function efficiently in high-salinity oilfield environments.

### 3.6 Cell-associated surface-active material effects on bacterial emulsification and hydrophilicity/hydrophobicity

**Fig. 4:**
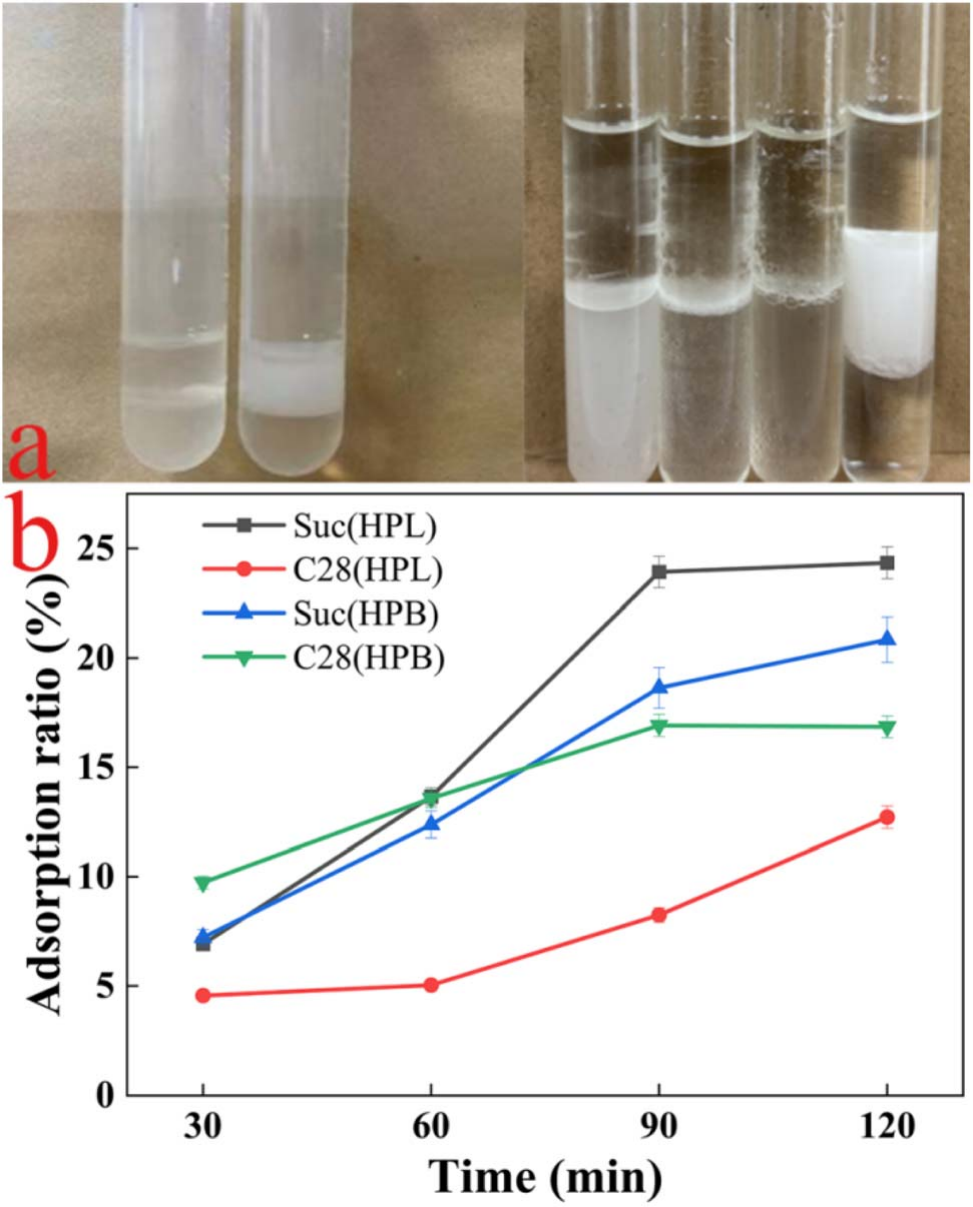
Cell surface emulsification by strain DH2. (a) (Left) Emulsification by the cell-free supernatant (left vial) and by the bacterial cells (right vial). (Right) Comparative emulsification by different biosurfactant-producing bacterial strains (from left to right): *Brevibacillus borstelensis*, *Pseudomonas aeruginosa*, *Bacillus subtilis*, and *Gordonia* sp. DH2. (b) Adsorption of strain DH2 to hydrophilic and hydrophobic quartz sand. Abbreviations: Suc (HPL/HPB), cell hydrophilicity/hydrophobicity from sucrose-based medium; C28 (HPL/HPB), cell hydrophilicity/hydrophobicity from C28-based medium.

In this study, surface-active material were synthesized by strain DH2 regardless of whether non-water-soluble or water-soluble carbon sources were provided as the sole carbon source. After centrifugation, emulsion tests of the supernatant and cell pellets revealed that emulsification activity was exclusively associated with the cell pellets, with no activity detected in the supernatant (Fig. 4a). In contrast, although they are biosurfactant-producing strains, the cell pellets of *Brevibacillus borstelensis*, *Pseudomonas aeruginosa*, and *Pseudomonas stutzeri* exhibited no discernible emulsifying activity. These observations suggest that the association of emulsifying activity with microbial cell surfaces is not a universal phenomenon. Rather than being secreted into the aqueous phase upon synthesis, the surface-active material remain adsorbed or entrapped on the cell envelope, thereby modulating the cell surface hydrophobicity. This surface modification is believed to enhance the intrinsic emulsifying activity of the microorganisms.

Adsorption equilibrium on hydrophilic/hydrophobic quartz sand was achieved within 90–120 min. Hydrophobicity analysis revealed differential adsorption patterns: 12.71% (Suc) and 20.84% (C28) of cells adhered to hydrophobic surfaces, *versus* 24.34% and 16.85% on hydrophilic surfaces, respectively (Fig. 4b). Therefore, hydrocarbon-mediated enhancement of cell surface hydrophobicity, while maintaining robust biosurfactant production across nutritional regimes. Further investigation is required to determine whether hydrophobic substrate contact induces surface-active compound production. Cell-associated surface-active material may facilitate biofilm formation at oil–water interfaces,^50^ enhancing hydrocarbon access. Similar mechanisms are observed in *Alcanivorax borkumensis*, where EPS-mediated biofilm formation reduces interfacial tension from 32 to 7 mN m^−1^,^51^ significantly improving degradation kinetics through interfacial restructuring. The hydrophobic cell surface architecture promotes direct hydrocarbon contact, minimizing mass transfer limitations and optimizing substrate uptake efficiency. This correlation between cell surface hydrophobicity and degradation capacity is well-documented.^52,53^

Most reported biosurfactants are water-soluble extracellular compounds, whereas cell-associated surface-active substances, as suggested in this work, have received comparatively limited attention. These surface-anchored surface-active material directly modify the physicochemical properties of microbial cells. Through this self-modification mechanism, bacterial adhesion to hydrophobic substrates, including oil droplets and organic pollutants-is significantly enhanced, promoting efficient substrate uptake and degradation.^54,55^ This represents an ecological strategy that is inherently “selfish,” as the resulting advantages are largely confined to the producing organism, thereby strengthening its competitive position in polymicrobial settings. Furthermore, owing to their firm association with cellular structures, these surface-active material exhibit considerable resistance to leaching and inactivation, even under challenging conditions such as high salinity or mineralization. In contrast, water-soluble extracellular biosurfactants are released into the ambient aqueous environment post-synthesis, where they act as diffusible surface-active agents. Their primary role lies in substantially lowering the oil–water interfacial tension within the medium, which indirectly increases the bioaccessible surface area of hydrophobic substrates. However, when employed in real-world applications-such as *in situ* bioremediation or microbial enhanced oil recovery-their functional performance is frequently compromised by environmental constraints, including dilution, sorption onto soil or mineral surfaces, and salt-induced precipitation or deactivation. *Gordonia* sp. is distinguished by its remarkable metabolic versatility toward hydrophobic substrates. Strain DH2 efficiently utilizes petroleum hydrocarbons for growth and biosurfactant production. These attributes enable direct reservoir inoculation for *in situ* surface-active material production from indigenous hydrocarbons, reducing crude viscosity and enhancing oil recovery while minimizing chemical surfactant costs. Strain DH2 concurrently degrades hydrocarbons and secretes surface-active material, facilitating oil emulsification and promoting syntrophic degradation with hydrocarbonoclastic consortia.^56^ The cell-associated surface-active material phenotype minimizes metabolic cross-feeding, conferring competitive advantage in mixed communities. This unique localization optimizes substrate acquisition efficiency by preventing surfactant dilution or nonspecific adsorption, while ensuring preferential resource allocation.

### 3.7 Hydrocarbons biodegradation by strain DH2 under high salinity

*Gordonia* sp. DH2 was isolated from an oilfield, and its genome suggests that it may possess the ability to degrade petroleum hydrocarbons. Therefore, it has been used to demonstrate hydrocarbon degradation capabilities under laboratory conditions. This study investigates the degradation of alkanes, aromatic hydrocarbons, DBT, and crude oil by *Gordonia* sp. DH2, analyzing the strain's characteristics in degrading petroleum hydrocarbons and crude oil.

#### 3.7.1 Degradation of alkanes

**Fig. 5:**
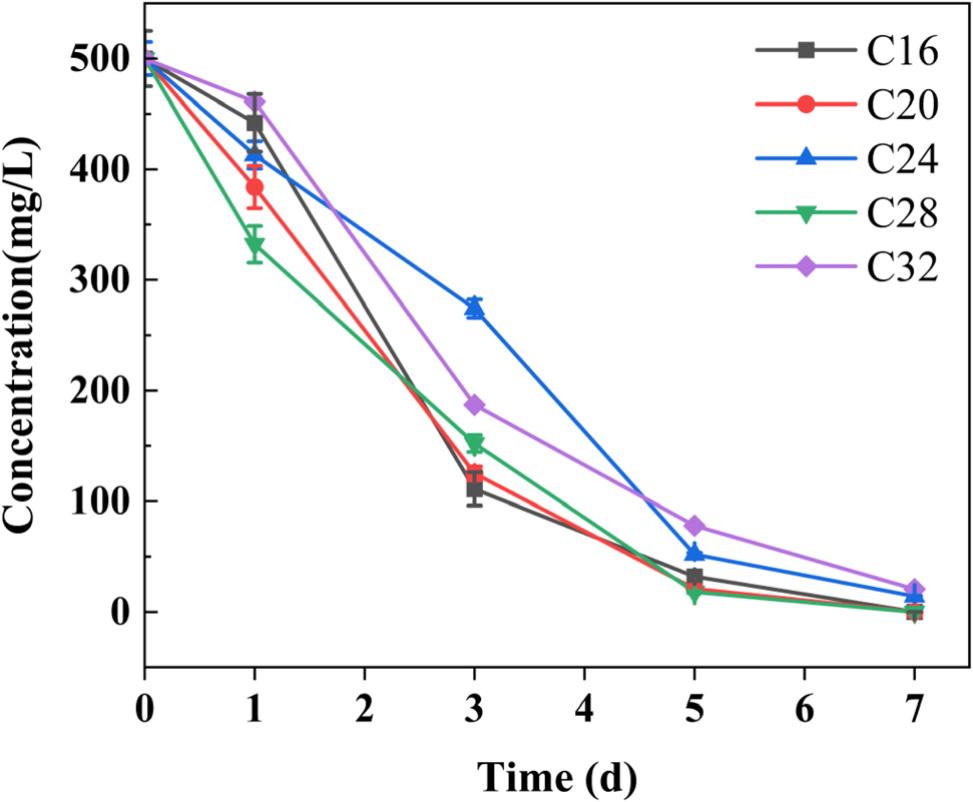
Degradation of alkane: (C16, C20, C24, C28, C32) by *Gordonia* sp. DH2 under 6% NaCl after 7 days of incubation. Values are expressed as mean ± SD of three independent experiments.

In the alkane degradation experiments, strain DH2 demonstrated remarkable degradation efficiency exceeding 95% for all tested alkanes (C16, C20, C24, C28, and C32) after 7 days of incubation. The initial concentration of 500 mg L^−1^ was reduced to residual levels (Fig. 5) of 0.1 mg L^−1^ (C16), 0.15 mg L^−1^ (C20), 14.21 mg L^−1^ (C24), and 20.6 mg L^−1^ (C32), with nearly complete degradation observed for C28. These results clearly indicate that strain DH2 exhibits remarkable degradation efficiency towards alkanes with carbon chain lengths ranging from C15 to C32, showing equally competent degradation capabilities for both medium/short-chain and long-chain hydrocarbons.

For medium and short-chain alkanes (C10–C20), degradation is primarily initiated by terminal oxidation catalyzed by *AlkB* type alkane monooxygenases, which introduce a hydroxyl group at the terminal carbon to produce corresponding primary alcohols. These alcohols are subsequently oxidized by alcohol dehydrogenases and aldehyde dehydrogenases to form fatty acids, which then enter the β-oxidation pathway for complete mineralization to CO_2_ and H_2_O. The presence of multiple *alkB* homologs in strain DH2 suggests a flexible and efficient enzymatic system for metabolizing a broad range of shorter-chain alkanes.

In contrast, medium- to long-chain alkanes (C20–C36) undergo oxidation mainly *via* the *LadA*-mediated terminal oxidation mechanism, in which the flavin-dependent monooxygenase *LadA* catalyzes the initial hydroxylation of the terminal methyl group. This reaction is particularly efficient for long hydrophobic substrates that are poorly accessible to membrane-bound *AlkB* enzymes. The *ladA* gene, identified in the genome of strain DH2, may encodes this heat-stable and membrane-associated enzyme,^33^ thereby compensating for the inherent substrate limitations of the conventional *AlkB* system and expanding the range of degradable hydrocarbons.

Under high-salinity conditions (6% NaCl, >1 M), the strain maintained robust adaptability and exceptional hydrocarbon degradation performance, which may be attributed to the coordinated expression of salt-tolerance genes (*nhaA*, *kefB*, *araJ*, *fucP*, *etc.*) and hydrocarbon-degrading genes. This unique genetic combination enables efficient alkane metabolism even in challenging hypersaline environments. Moreover, surface-active material can enhance microbial degradation of petroleum hydrocarbons (including *n*-alkanes and aromatic hydrocarbons).^57^*Gordonia* sp. SBUG 1971 and SUBG 1972 have been documented to degrade 24 *n*-alkanes, 22 *n*-alkylcyclohexanes, and 20 branched alkanes.^58^ Furthermore, strain DH2 demonstrates biosurfactant production capability when grown on alkanes as the sole carbon source, facilitating substrate emulsification and uptake. These results indicate that strain DH2 showed strong degradation capacity toward alkanes ranging from C16 to C32 under the tested conditions. The observed phenotype is consistent with the genome-predicted presence of alkB- and ladA-related genes. In addition, the cell-associated surface-active material observed in DH2 may have facilitated hydrocarbon contact and uptake. Together, these findings suggest that *Gordonia* sp. DH2 possesses substantial alkane biodegradation potential under saline conditions.

#### 3.7.2 Degradation of aromatic hydrocarbons and desulfurization performance

After 7 days of cultivation, strain DH2 demonstrated complete degradation (100%) of naphthalene and a 16.45% degradation rate for pyrene. These results clearly indicate that polycyclic aromatic hydrocarbons (PAHs) with fewer aromatic rings (such as naphthalene) are more readily degraded compared to those with more rings (pyrene). This observation aligns with the general principle that microbial degradation efficiency typically decreases as the number of aromatic rings in PAHs increases.

*Gordonia* sp. has been widely reported for its PAH degradation capabilities. Several species, including *Gordonia paraffinivorans*, *Gordonia alkanivorans*, and *Gordonia terrae*,^59,60^ have been confirmed to degrade PAHs in crude oil. Most of these bacteria employ extracellular enzymes (cytochrome P450 and alkane monooxygenases) to catalyze the oxidation of PAHs,^61^ facilitating their breakdown. For comparison, under similar conditions, *Gordonia alkaliphila* JCM 18077T and *Gordonia paraffinivorans* DSM 44604T exhibited naphthalene degradation rates of 70.5% and 89.8%, respectively.^62^ This study confirms that *Gordonia* sp. DH2 efficiently degrades naphthalene among aromatic hydrocarbons and possesses significant potential for bioremediation of PAH-contaminated water and soil.

**Fig. 6:**
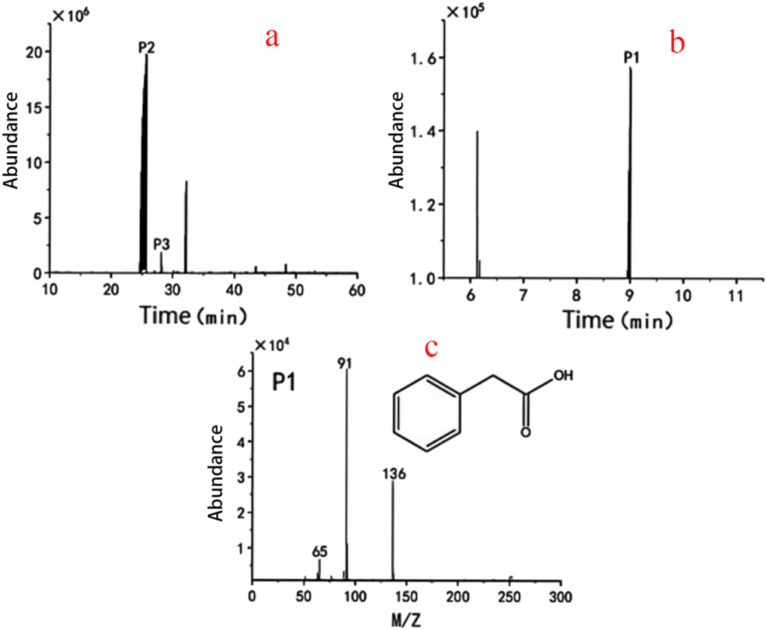
GC-MS analysis of DBT degradation by *Gordonia* sp. DH2. (a) Total ion chromatogram of metabolites; (b) extracted ion chromatogram of P1; (c) mass spectrum of P1 identified as phenylacetic acid.

DBT is a fused polycyclic aromatic hydrocarbon among aromatic hydrocarbons, considered the most challenging sulfur compound to desulfurize in diesel fractions, and serves as an important model compound in diesel hydrodesulfurization research. Therefore, the content of DBT is often used to evaluate the sulfur content of fuels and the efficiency of desulfurization. In the quantitative analysis using GC-MS, 4-methylDBT was added as an internal standard to calculate the degradation rate of DBT by *Gordonia* sp. DH2. The peak P2 at a retention time of 25.61 min had a mass-to-charge ratio (*m*/*z*) of 184 and contained ion fragments with *m*/*z* of 158, 139, and 92 (Fig. 6a), identifying this substance as DBT (Table S4). The peak P3 at a retention time of 28.08 min had a mass-to-charge ratio (*m*/*z*) of 198 and contained ion fragments with *m*/*z* of 165, 151, and 98. The concentration of DBT decreased from an initial 100 mg L^−1^ to 0.95 mg L^−1^, achieving a degradation rate of 99%. Thus, strain DH2 exhibits excellent degradation capability for DBT while also possessing desulfurization functionality.

In the degradation products of DBT, the product peak P1 at a retention time of 8.99 min had a mass-to-charge ratio (*m*/*z*) of 136 and contained ion fragments with *m*/*z* of 91 and 65. This substance was identified as phenylacetic acid (Fig. 6b and c). This compound does not contain sulfur, indicating that *Gordonia* sp. DH2 has excellent desulfurization capability. According to current literature,^63^ the desulfurization by *Gordonia* sp. primarily occurs *via* the 4S pathway, with the desulfurized product being 2-hydroxybiphenyl. DBT serves only as a sulfur source, not as a carbon source.

## 4 Conclusion

A newly isolated strain, *Gordonia* sp. DH2, was able to utilize petroleum hydrocarbons as sole carbon sources and exhibited strong cell-associated emulsifying activity. The extracted cell-associated surface-active material showed glycolipid-like characteristics based on TLC and FTIR analyses, although its exact chemical structure remains to be further resolved. This surface-active behavior appears to contribute to the modification of cell surface properties and to enhanced interfacial interaction with hydrophobic substrates, thereby supporting efficient hydrocarbon utilization.

## Conflicts of interest

There are no conflicts of interest to declare.

## Supplementary Material

RA-016-D5RA09392A-s001

## Data Availability

All data generated or analysed during this study are included in the supplementary information (SI). Supplementary information is available. See DOI: https://doi.org/10.1039/d5ra09392a.
