# Supplementary material for: Halotolerant Gordonia sp. DH2 enhances hydrocarbon degradation and emulsification in high-salinity oil reservoirs through cell-associated surface-active material
Source: RSC Adv. 2026 Apr 28;16(24):21942–52. doi: 10.1039/d5ra09392a (PMC13123891; doi:10.1039/d5ra09392a)
Supplement: RA-016-D5RA09392A-s001 [file RA-016-D5RA09392A-s001.pdf]

Supplementary Information (SI) for RSC Advances.
This journal is © The Royal Society of Chemistry 2026

Halotolerant *Gordonia sp*. DH2 Enhances Hydrocarbon Degradation and Emulsification in High-Salinity Oil Reservoirs through cell-bound Biosurfactant Production

Ji Gao[1], Yu Duan[1], Ting Xu[2], Ding Dong[3], Dehao Lu[4], Shuyuan Deng[5], Jian Fu[5], Fan Zhang[6], Hao Dong[1, *], Yuehui She[5, *]

[1] Hubei Engineering Research Centers for Clean Production and Pollution Control of Oil and Gas Fields, College of Chemistry and Environmental Engineering, Yangtze University, Jingzhou 434023, China

[2]College of Resources and Environment, Yangtze University, Wuhan, Hubei 430010, China

[3]Karamay Xinaoda Petroleum Technological Co., Ltd, Xinjiang, 83400, China

[4]Xinjiang Oilfield Company PetroChina, Karamay, Xinjiang, 834000, China

[5]College of Petroleum Engineering, Yangtze University, Wuhan, Hubei 430010, China

[6]The Key Laboratory of Marine Reservoir Evolution and Hydrocarbon Accumulation Mechanism, Ministry of Education, College of Energy Resources, China University of Geosciences (Beijing), Beijing 100083, China

*** Correspondence:** *Corresponding author.

E-mail: dong_hao2005@163.com; sheyuehui@163.com

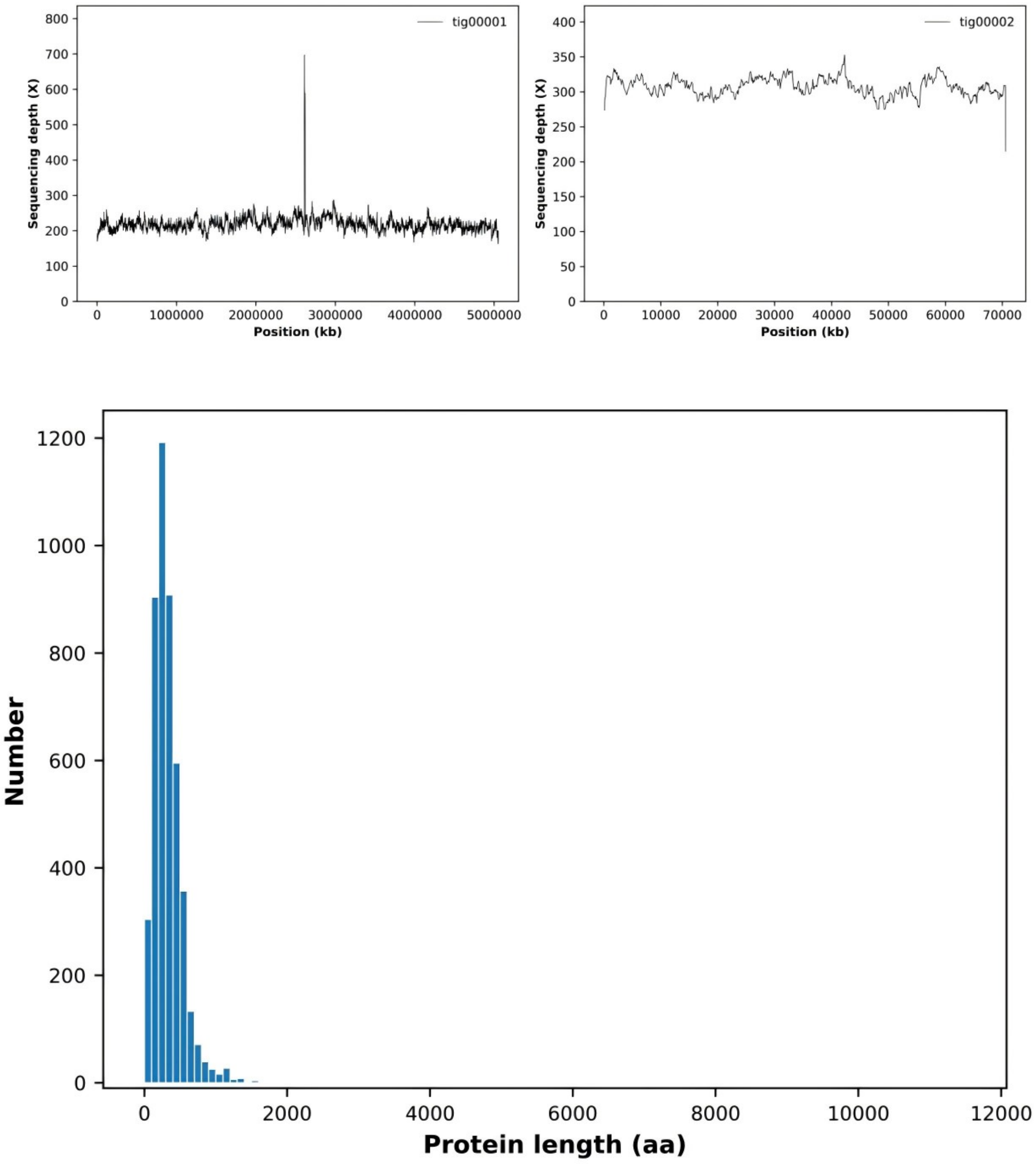

Fig. S1. (a) Distribution of genome sequencing depth (window=1000 bp); (b) Distribution of encoded protein lengths (window=100 aa).

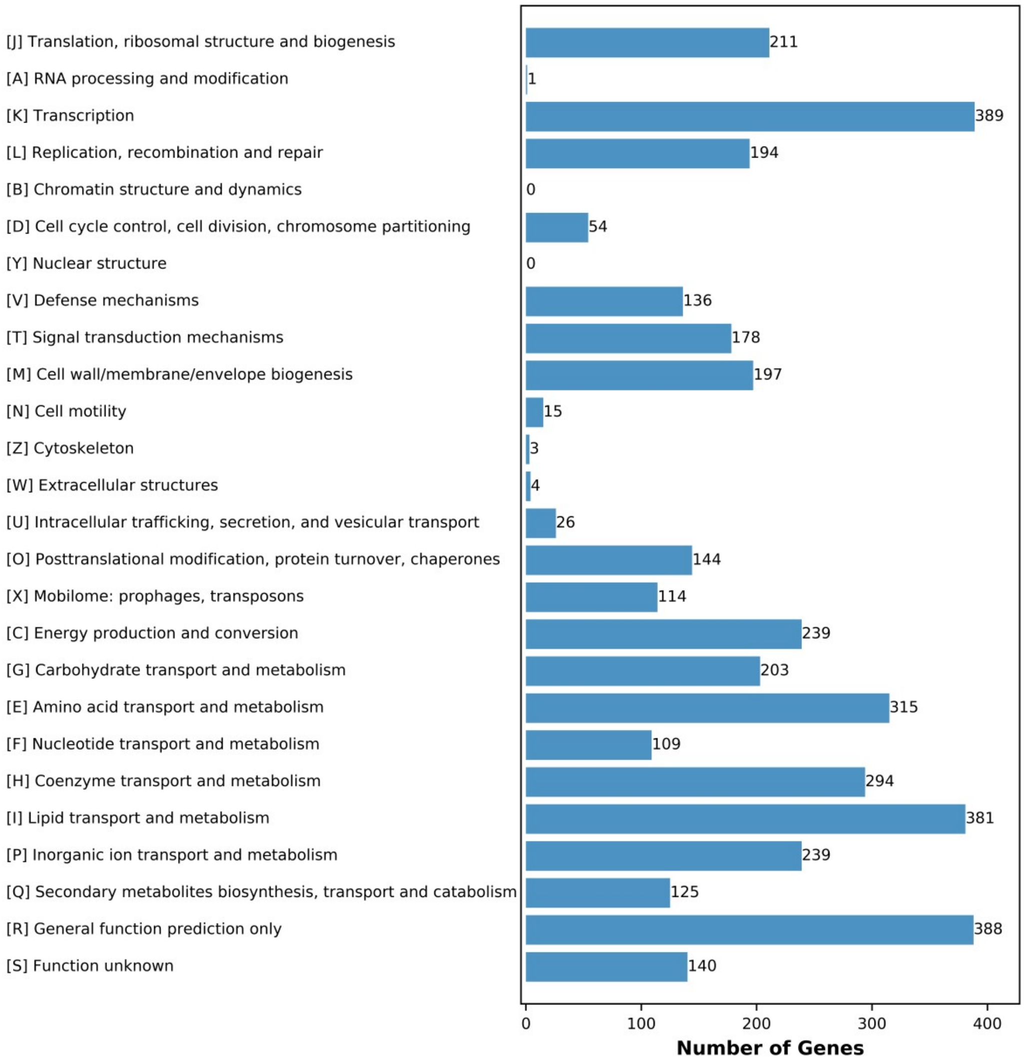

Fig. S2. COG functional classification statistics of encoded proteins in the genome.

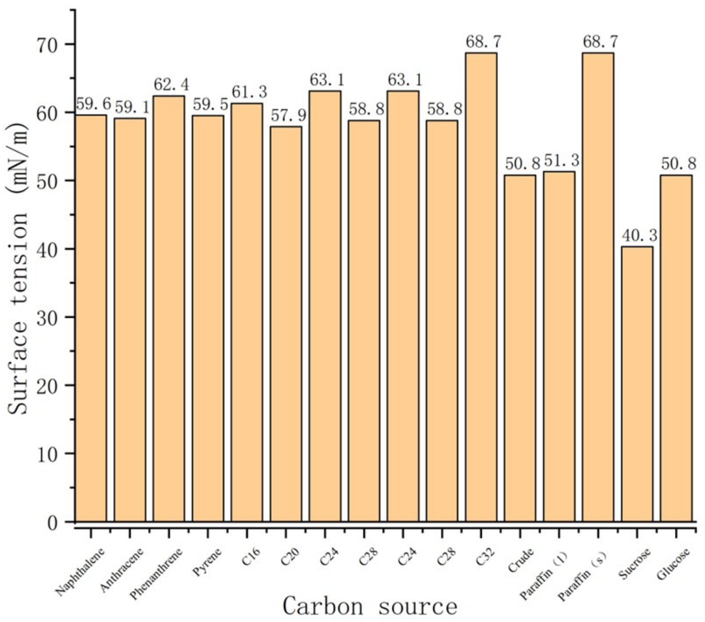

Fig. S3 Surface Tensions of Different Carbon Sources

The genome consists of a circular chromosome of 5,054,758 bp with a GC content of 67.38%, containing 4,604 predicted coding sequences (CDSs) (Fig S1b), 51 tRNAs, and 12 rRNAs (4 copies of 16S rRNA, 4 copies of 23S rRNA, and 4 copies of 5S rRNA) (Table S1). Additionally, the genome includes a 70,604 bp plasmid. In Fig S2, 389 genes are involved in transcription, 239 genes are related to energy production and conversion, and 315 genes are allocated for amino acid transport and metabolism. These findings suggest the high metabolic capacity of *Gordonia* sp. DH2, its potential to thrive in extreme environments, and its capability for biosurfactants production.

Tab. S1. Statistics of genomic structure prediction.

Type	Number	Length (bp)	% genome
tRNA	51	3,879	0.08
rRNA (16S)	4	6,090	0.12
rRNA (23S)	4	12,540	0.24
rRNA (5S)	4	412	0.01
CDS	4,604	4,671,234	91.14
CRISPR	0	0	0
genomic_island	15	412,240	8.04
prophage_region	2	52,032	1.02

Tab. S2 Halotolerance genes within the genome of strain DH2.

Gene	Function
nhaA	Na^+/H^+ antiporter NhaA
nhaP	NhaP-type Na^+/H^+ or K^+/H^+ antiporter
kefB	Kef-type K^+ transport system%2C membrane component KefB
plsC	Major Facilitator Superfamily
sulP	Sulfate permease or related transporter%2C MFS superfamily
gluP	Major Facilitator Superfamily
fucP	Major Facilitator Superfamily
gph	MFS/sugar transport protein
sul1	Sulfate permease or related transporter%2C MFS superfamily
araJ	Major Facilitator Superfamily
trkH	Trk-type K^+ transport system%2C membrane component
trkA	Ion transport protein
nhaP	NhaP-type Na^+/H^+ and K^+/H^+ antiporter with C-terminal TrkAC and CorC domains
kdpD	K^+-sensing histidine kinase KdpD
gltD	NADPH-dependent glutamate synthase beta chain or related oxidoreductase
gltB1	Glutamate synthase central domain
betB	Choline-glycine betaine transporter
betA	Choline dehydrogenase or related flavoprotein
glnA	Glutamine synthetase
etcA	Synthesis of ectoine
etcB	Synthesis of ectoine
etcC	Synthesis of ectoine
proB	Synthesis of proline
metlD	Synthesis of mannitol
CAT1	Decomposition of H_2O_2
HSP70	Prevention of protein denaturation under salt stress
AQP	Regulation of water permeability
MscS	Osmotic shock protection

Tab. S3 Biosurfactant-producing genes and gene clusters within the genome of strain DH2.

Gene	*Function*
EntF	*Non-ribosomal peptide synthetase*
EntF2	*Non-ribosomal peptide synthetase*
PapA3	*PKS-related acyltransferase*
SrfAC	*Surfactin synthetase complex component*
FenE	*Fengycin synthetase。*
RfaB	*Glycosyltransferase involved in lipopolysaccharide core synthesis*
WcaA	*Polyprenol phosphate mannose synthase, catalyzing mannosyl transfer in glycolipids*
wax	*Trehalose dimycolate (TDM) synthase, producing trehalose lipid surfactants*
YjhB	*Mannosyltransferase, involved in glycosyl modification of glycolipids*
GlfT1	*Galactosyltransferase involved in galactolipid synthesis*
GlfT2	*Galactosyltransferase involved in galactolipid synthesis*
FabG	*Providing precursors of fatty acid chains for surfactants。*
AcpP	*Carrying fatty acid chains for glycolipid synthesis*
FadD	*Activation of fatty acid chains*
ComP	*Regulation of biosurfactant synthesis gene cluster expression*
DrrA	*Involvement in biosurfactant secretion*
DrrB	*Involvement in biosurfactant secretion*

Tab. S4 Hydrocarbon-degrading genes within the genome of strain DH2.

Gene	*Function*
alkB	*Catalytic terminal hydroxylation of alkanes*
LadA	*Degradation of long-chain alkanes*
AlmA	*Degradation of long-chain alkanes*
EthA	*Catalytic initial oxidation of short-chain alkanes*
EthB	*Catalytic initial oxidation of short-chain alkanes*
BenA	*Catalytic dihydroxylation of benzene ring*
BenB	*Catalytic dihydroxylation of benzene ring*
XylA	*Participation in toluene/xylenes metabolic pathway*
XylB	*Participation in toluene/xylenes metabolic pathway*
DmpK	*Degradation of phenol and its derivatives*
DmpB	*Degradation of phenol and its derivatives*
LeuA	*Degradation of phenol and its derivatives*
mhpF	*Degradation of phenol and its derivatives*
dmpG	*Degradation of phenol and its derivatives*
mhpE	*Degradation of phenol and its derivatives*
PhnA	*Catalytic initial oxidation of naphthalene, phenanthrene*
PhnB	*Catalytic initial oxidation of naphthalene, phenanthrene*
NidA	*Pyrene-degrading dioxygenase*
NidB	*Pyrene-degrading dioxygenase*
NirB	*Pyrene-degrading dioxygenase*
hcaD	*Pyrene-degrading dioxygenase*
PaaJ	*Degradation of phenylacetic acid*
PaaD	*Degradation of phenylacetic acid*
paaF	*Degradation of phenylacetic acid*
PaaK	*Degradation of phenylacetic acid*

beta	*Degradation of phenylacetic acid*
Fdx	*Degradation of phenylacetic acid*
FadD	*Participation in β-oxidation of alkane derivatives*
FadL	*Participation in β-oxidation of alkane derivatives*
Cypx	*Oxidation of both aromatic and aliphatic hydrocarbons*
AdhE	*Oxidation of alkane-derived primary alcohols to carboxylic acids*

Tab. S5 Metabolites of dibenzothiophene detected by GC-MS.

Product peak	Retention time（min）	MW and m/z	Metabolic intermediates
P1	8.99	136（136,91,65）	Phenylacetic acid
P2	25.61	184（184, 158,139,92）	DBT
P3	28.08	198（198,165,151,98）	4-MDBT
